# Role of SUV39H2 in shaping the malignant phenotype of triple-negative breast cancer

**DOI:** 10.3389/fonc.2025.1679640

**Published:** 2026-01-02

**Authors:** Zhi-Wen Wang, Shuang Hu, Yong Chen, Yong Lu, Shang-Fu Li, Yang Deng, Hai-Tao Jiang

**Affiliations:** 1 Key Laboratory of Chronic Noncommunicable Diseases, Yueyang Vocational Technical College, Yueyang, Hunan, China; 2 Institute of Biology and Medicine, College of Life and Health Sciences, Wuhan University of Science and Technology, Wuhan, Hubei, China; 3 Yueyang People’s Hospital, Yueyang Hospital Affiliated to Hunan Normal University Department of Anesthesiology, Yueyang, Hunan, China; 4 College of Life Sciences, Jianghan University, Wuhan, Hubei, China

**Keywords:** SUV39H2, TNBC, diagnostic biomarker, BRCA subtypes, TCGA

## Abstract

**Background:**

SUV39H2, a histone methyltransferase involved in H3K9 trimethylation and transcriptional silencing, has been implicated in cancer progression. However, its expression patterns, clinical relevance, and functional roles in various tumor types remain to be fully elucidated.

**Methods:**

SUV39H2 expression in multiple cancers and paired adjacent normal tissues was analyzed using the TCGA-GTEX database and Xiantao online tools. Prognostic significance was assessed by survival analysis. KEGG and GO enrichment analyses were conducted to explore potential functional roles. Immune infiltration correlations were evaluated based on TCGA data. Single-cell RNA sequencing from GSE176078 was analyzed for validation of expression patterns. Immunohistochemistry (IHC) was performed on five pairs of TNBC patient samples to confirm SUV39H2 expression. Western blotting (WB) was conducted to verify SUV39H2 knockdown efficiency in MDA-MB-231 and Hs-578T TNBC cells. Functional assays, including Transwell migration, colony formation, sphere formation, and subcutaneous xenograft tumorigenicity assays, were performed to evaluate the effects of SUV39H2 knockdown on TNBC cell growth and metastatic potential.

**Results:**

SUV39H2 was significantly overexpressed in multiple cancers, with consistently elevated levels in paired tumor-adjacent tissues. In TNBC, SUV39H2 expression was markedly upregulated across all clinical and TNM stages compared with normal tissues and other BRCA subtypes. Immunohistochemistry confirmed SUV39H2 overexpression in TNBC patient samples, and WB demonstrated successful knockdown in MDA-MB-231 and Hs-578T cells. Functional assays revealed that SUV39H2 knockdown significantly inhibited proliferation, migration, stemness, and *in vivo* tumorigenicity in TNBC cells.

**Conclusions:**

SUV39H2 is consistently upregulated in triple-negative breast cancer and is associated with malignant features including enhanced proliferation, migration, stemness, and *in vivo* tumorigenicity. Functional and clinical evidence highlights SUV39H2 as a potential diagnostic biomarker and therapeutic target in TNBC, although its clinical utility requires further validation.

## Introduction

Breast cancer (BRCA) remains a formidable global health burden, characterized by intricate pathogenesis and pronounced clinical heterogeneity, which continue to impede the development of effective therapeutic and preventive strategies (1–3). Its diverse molecular subtypes exhibit distinct epidemiological profiles, contributing to diagnostic and treatment complexity (4). Globally, breast cancer accounts for nearly one-third of all newly diagnosed female malignancies and approximately 15% of cancer-related deaths in women (5, 6). This persistent global rise in incidence is driven by the multifactorial interplay of genetic predisposition, environmental exposures, and lifestyle factors (5). Accordingly, elucidating the multifaceted biology of breast cancer is vital for the formulation of precision oncology approaches.

Triple-negative breast cancer (TNBC), a particularly aggressive subtype, exemplifies the challenge of intratumoral heterogeneity and frequent therapeutic resistance. The lack of robust biomarkers and high clonal diversity remain key barriers to durable treatment responses (7). Representing 15%–20% of all breast cancer cases, TNBC is defined by the absence of estrogen receptor (ER), progesterone receptor (PR), and HER2 overexpression or amplification (8). Compared to hormone receptor-positive or HER2-driven breast cancers, TNBC exhibits earlier onset, greater metastatic potential, and markedly poorer prognosis, with elevated relapse rates and shortened survival times (9, 10). Although some patients with early-stage TNBC benefit from chemotherapy, the overall survival (OS) for those with metastatic disease remains dismal, ranging from 13 to 18 months under current treatment paradigms (11).

Despite curative-intent surgery and absence of initial metastasis, nearly half of TNBC patients eventually experience recurrence (12). Although the pathological complete response (pCR) rate following neoadjuvant chemotherapy is higher in TNBC (22–45%) compared to other subtypes (~7%) (13), this advantage does not translate into survival benefit, as mortality remains disproportionately elevated. Unlike ER- or HER2-positive breast cancers, TNBC lacks approved molecularly targeted therapies, rendering systemic chemotherapy the prevailing standard. However, its benefit is often transient, and long-term disease control remains elusive.

On clinical, histopathological, and molecular levels, TNBC is remarkably heterogeneous (14). Genomic instability and elevated mutational burdens are hallmarks of this subtype, potentially enhancing tumor immunogenicity through increased neoantigen presentation (15, 16). TNBC is widely regarded as the most immunoreactive breast cancer subtype, with heightened PD-L1 expression and enriched tumor-infiltrating lymphocytes (TILs) (17). However, its tumor immune microenvironment (TIME) is complex and variable, comprising diverse cellular populations and exhibiting uneven gene expression patterns (18). High proliferative activity often gives rise to hypoxia and necrosis within the tumor, which in turn reshapes the immunogenomic landscape, influencing immune cell viability, tumor recognition, and antitumor responses (19, 20).

A comprehensive understanding of breast cancer pathogenesis and its molecular taxonomy is pivotal for anticipating disease progression, therapeutic response, and patient outcomes. Such insights are essential for designing personalized therapies aimed at improving clinical prognosis and quality of life (21).

The histone methyltransferase SUV39H2 (Suppressor of Variegation 3–9 Homolog 2, also known as KMT1B), a member of the SUV39 subfamily of lysine methyltransferases, predominantly localizes to the nucleus and catalyzes di- and trimethylation of histone H3 at lysine 9 (H3K9) (22, 23). The resultant H3K9me3 mark plays a critical role in establishing transcriptionally silent heterochromatin, particularly in pericentromeric and telomeric domains, and contributes to the epigenetic silencing of euchromatic regions (24–28).

Three alternative splice isoforms of SUV39H2 have been described, with exon 3 influencing its enzymatic activity, stability, and nuclear localization (29). SUV39H2 interacts with retinoblastoma (Rb) family pocket proteins, facilitating repression of E2F target genes through H3K9me3 enrichment at their promoters and regulating cell cycle progression and differentiation (30, 31). Repressive chromatin configurations marked by H3K9me3 are also enriched at p53-responsive loci, modulating apoptosis and cell proliferation pathways (32).

In murine MMTV-PyMT models of breast cancer, SUV39H2 and other histone methyltransferases are upregulated at metastatic lung sites compared to primary tumors and disseminated cells (33). Clinically, SUV39H2 is highly expressed in basal-like breast cancer and correlates with adverse prognosis in patients (34). Emerging evidence suggests that dysregulation of SUV39H2 contributes to aberrant histone methylation signatures, driving breast cancer progression (35). During metastasis, distinct epigenetic programs are reconfigured, yielding subtype-specific biomarkers. As SUV39H2 is implicated in hormone-responsive regulation and metastatic potential, it may serve as a prognostic indicator and therapeutic target in TNBC and advanced-stage breast cancer (36).

## Materials and methods

### Analysis of SUV39H2 expression and its prognostic value across cancer types

To investigate the expression profile of SUV39H2 across cancers, we utilized the Xiantao platform (https://www.xiantao.love/) to generate a pan-cancer expression landscape, including paired comparisons between tumor and adjacent normal tissues. The version of R used in the website is R (4.2.1), and the R packages utilized are: ggplot2 [3.4.4], stats [4.2.1], and car [3.1-0]. Statistical analysis was performed using the Wilcoxon rank sum test. Expression data were obtained from the TCGA database (https://portal.gdc.cancer.gov) where RNAseq data from 33 tumor projects processed by the STAR pipeline were downloaded and organized. The TPM format data were extracted, and the data processing method involved log2(value + 1).

The full names of all tumors are detailed in **Supplementary Table S1**. We specifically analyzed adrenocortical carcinoma (ACC), breast invasive carcinoma (BRCA), and liver hepatocellular carcinoma (LIHC), evaluating SUV39H2 mRNA levels in both normal and tumor-adjacent samples. The analysis method for individual tumors was consistent with the above analysis.

We subsequently assessed the prognostic significance of SUV39H2 expression using survival metrics derived from The Cancer Genome Atlas (TCGA), including overall survival (OS), progression-free interval (PFI), and disease-specific survival (DSS) in ACC (n=79 for OS/PFI, n=77 for DSS), BRCA (n=1086/1066), LIHC (n=373/365), and sarcoma (SARC) (n=263/257). In this step, the R packages used were: survival [3.3.1], survminer [0.4.9], and ggplot2 [3.4.4]. The statistical method applied was Cox regression. Data were obtained from the TCGA database, where RNAseq data from the TCGA projects (ACC, BRCA, LIHC, and SARC) processed by the STAR pipeline were downloaded and organized, along with clinical data. The data processing method was consistent with the one described above. In parallel, receiver operating characteristic (ROC) curve analyses were performed to evaluate the diagnostic potential of SUV39H2. In this step, the R packages used were: pROC [1.18.0] and ggplot2 [3.4.4]. The source of expression data and the data processing method were consistent with the above methods. Subgroup comparisons were conducted within the BRCA and LIHC cohorts to determine statistical associations between SUV39H2 expression and clinical variables including age, T stage, and molecular subtype. In this step, the R packages used were: ggplot2 [3.4.4], stats [4.2.1], and car [3.1-0]. The statistical method applied was the Kruskal-Wallis test. The source of expression data and the data processing method were consistent with the above methods. To further explore SUV39H2 expression patterns in breast cancer, especially in TNBC, we employed the Home-for-Researchers platform (https://www.home-for-researchers.com/#/) to conduct dimensionality reduction and clustering analysis on the GSE176078 dataset. In this step, Download the corresponding single-cell data in.h5 format and annotation results from TISCH. Use the R software MAESTRO and Seurat to process and analyze the single-cell data. Re-cluster the cells using the t-SNE method.

Additionally, R software (version 4.1.0), developed by George Ross Ihaka and Robert Gentleman (Auckland, New Zealand), was used to process and filter TNBC-specific data from TCGA. We compared SUV39H2 expression between 114 TNBC tumors and 112 normal samples, and analyzed differences in expression across clinical stages and TNM classifications. Among the 115 TNBC samples, Pathological stage (I: 17, II: 68, III: 16, IV:2), Pathological T stage (T1: 23, T2: 70, T3: 10, T4:3), Pathological N stage (N0: 47, N1: 22, N2: 11), Pathological M stage (M0: 89, M1: 2, MX: 14). Among the 998 BRCA samples, Pathological stage (I: 160, II: 503, III: 196, IV:16), Pathological T stage (T1: 242, T2: 509, T3: 105, T4:29), Pathological N stage (N0: 261, N1: 127, N2: 99), Pathological M stage (M0: 740, M1: 17, MX: 124). In this step, the R packages used, statistical methods, data source, and data processing method were all consistent with the above methods.

### KEGG and GO enrichment analysis

To further elucidate the biological functions of SUV39H2, we performed Gene Ontology (GO) and Kyoto Encyclopedia of Genes and Genomes (KEGG) pathway enrichment analyses using TCGA-BRCA and TCGA-LIHC datasets via the Xiantao platform (https://www.xiantao.love/). The R package used was ggplot2 [3.4.4].

### Correlation between SUV39H2 expression and immune infiltration

To investigate the potential immunological role of SUV39H2, we constructed a correlation model between SUV39H2 expression and immune responses using the Xiantao academic platform (https://www.xiantao.love/). A pan-cancer landscape was generated to visualize the association between SUV39H2 and various immune cell populations. Furthermore, we performed immune infiltration analyses in BRCA and LIHC cohorts, assessing the relationship between SUV39H2 expression and the infiltration levels of distinct immune cell subsets.

### *In vitro* functional assays

The experimental procedures for the colony formation, sphere formation, and Transwell assays were conducted as previously described (38), with minor modifications. MDA-MB-231 cells were cultured for 10 days in standard medium for the colony formation assay. For the sphere formation assay, cells were maintained under low-attachment conditions for 10 days to allow spheroid development. In the Transwell assay, MDA-MB-231 cells were seeded into the upper chambers and incubated for 48 hours to assess migratory and invasive capacity.

### Western blot

The experimental procedures for the Western Blot was conducted as previously described (38). SUV39H2 antibody (11338-1-AP, China, wuhan, Proteintech, 1:2000), GAPDH antibody (60004-1-Ig, China, wuhan, Proteintech, 1:50000), AKT antibody (60203-2-Ig, China, wuhan, Proteintech, 1:1000), p-AKT antibody (66444-1-IG, China, wuhan, Proteintech, 1:1000).

### Cell culture

The MDA-MB-231 and Hs-578T cell lines were obtained from Wuhan Hualmei Bioengineering Co., Ltd. (Wuhan, China) and authenticated by the supplier. Cells were cultured in Dulbecco’s Modified Eagle Medium (DMEM) supplemented with 10% fetal bovine serum (FBS, Hyclone) and 1% penicillin–streptomycin, and maintained at 37°C in a humidified incubator with 5% CO_2_. The medium was changed every 2–3 days, and cells were passaged at approximately 80–90% confluency using 0.25% trypsin-EDTA.

### Stable SUV39H2 knockdown cell line construction

HEK-293T cells in logarithmic growth phase were seeded into 10 cm dishes at a density of 5 × 10^6^ cells/mL. Lentiviral particles were generated by co-transfecting SUV39H2 shRNA plasmids, GAG plasmid, and VSVG envelope plasmid (all from Invitrogen, USA) using Lipofectamine™ 2000 (Invitrogen, USA), according to the manufacturer’s instructions. After 24 hours, the transfection medium was replaced with fresh DMEM. The viral supernatants were collected at 72 hours post-transfection, filtered through a 0.45 μm membrane, aliquoted, and stored at −80°C. Viral titers were determined using the Lenti-Pac™ HIV RT-qPCR Titration Kit (GeneCopoeia, USA). For stable cell line generation, MDA-MB-231 and Hs-578T cells were infected with the prepared lentiviral supernatants and incubated at 37°C with 5% CO_2_ (Thermo Fisher, USA) for 72 hours. Subsequently, cells were selected with puromycin (2 μg/mL) for 3 days. Surviving cells were expanded and validated for subsequent experiments. Endotoxin-free plasmids were extracted using the Endotoxin-Free Plasmid Extraction Kit (CWBio, Beijing, China), and transfections were carried out using Lipofectamine™ 2000 (Invitrogen, USA). The shRNA sequence of SUV39H2 is as follows: sh-SUV39H2-1: 5’-GGGAAGAGTATGTTGAAATTT-3’; sh-SUV39H2-2: 5’-GGAGTTGTGGAATTCAGATTT-3’.

### Nude mouse xenograft model

The experimental procedures for the Nude Mouse Xenograft Model were conducted according to the previous experimental methods (37). Four-week-old female BALB/c nude mice were used. Tumor tissues were harvested, weighed, and photographed after euthanasia at 28 days injection. Tumor volume was measured weekly using the formula: Volume = (length × width²)/2. No volume measurements were performed in the first two weeks, as no obvious tumors had formed during this period.

### Immunohistochemistry

Formalin-fixed, paraffin-embedded tissue sections from clinical breast cancer specimens were obtained from Fabio Biotechnology Co., Ltd. (Wuhan, China). Sample processing and staining were performed according to the company’s standardized immunohistochemical protocol.

### Statistical analyses

The experimental procedures for the Statistical Analyses was conducted as previously described (37).

## Results

### Expression and prognostic significance of SUV39H2 in multiple tumor types

**Figure 1:**
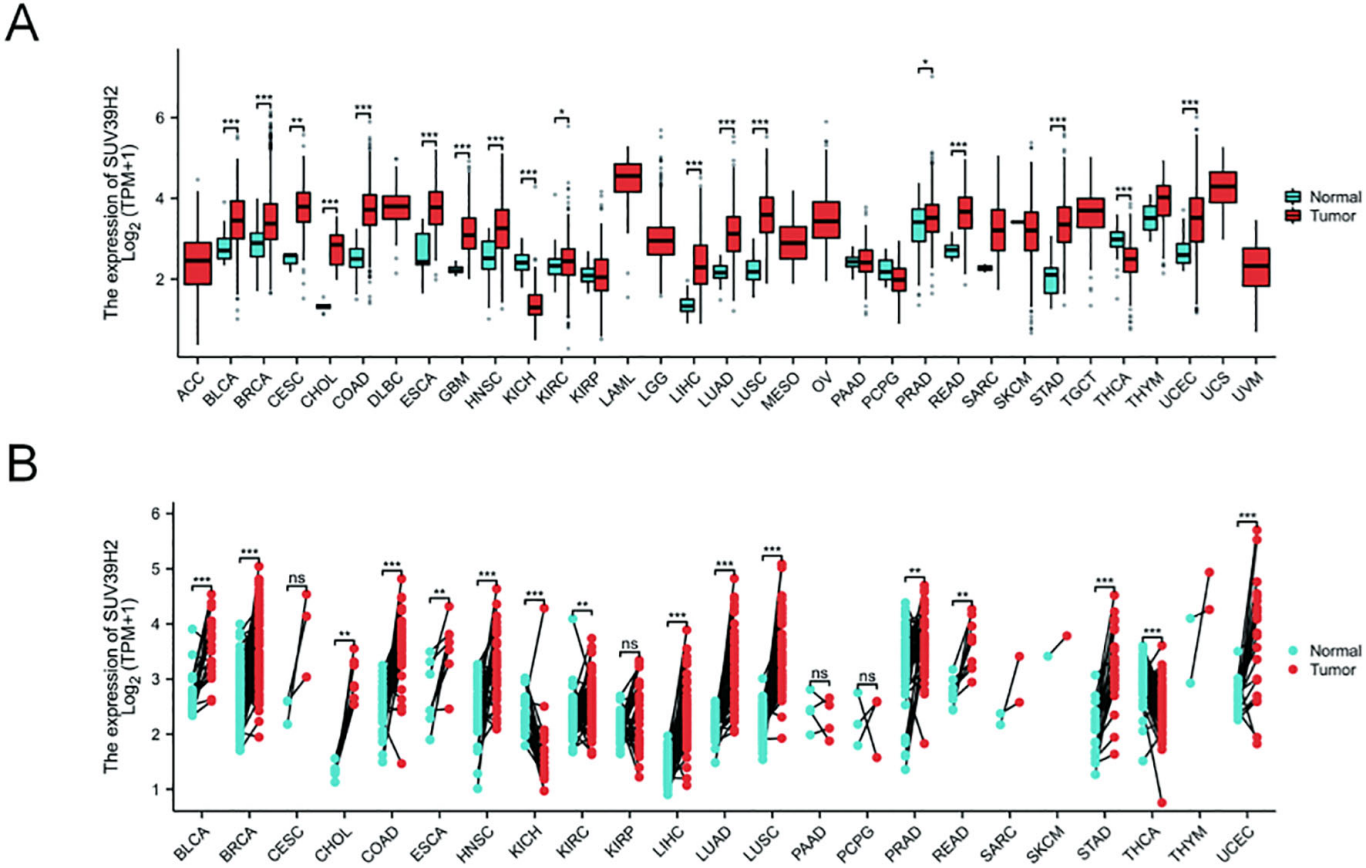
SUV39H2 is highly expressed in multiple cancers. **(A)** Pan-cancer analysis of SUV39H2 mRNA expression in tumor versus normal tissues based on TCGA-GTEx data. **(B)** Paired comparison of SUV39H2 expression between tumor and adjacent normal tissues in TCGA cohorts. P < 0.05 (*), P < 0.01 (**), P < 0.001 (***).

**Figure 2:**
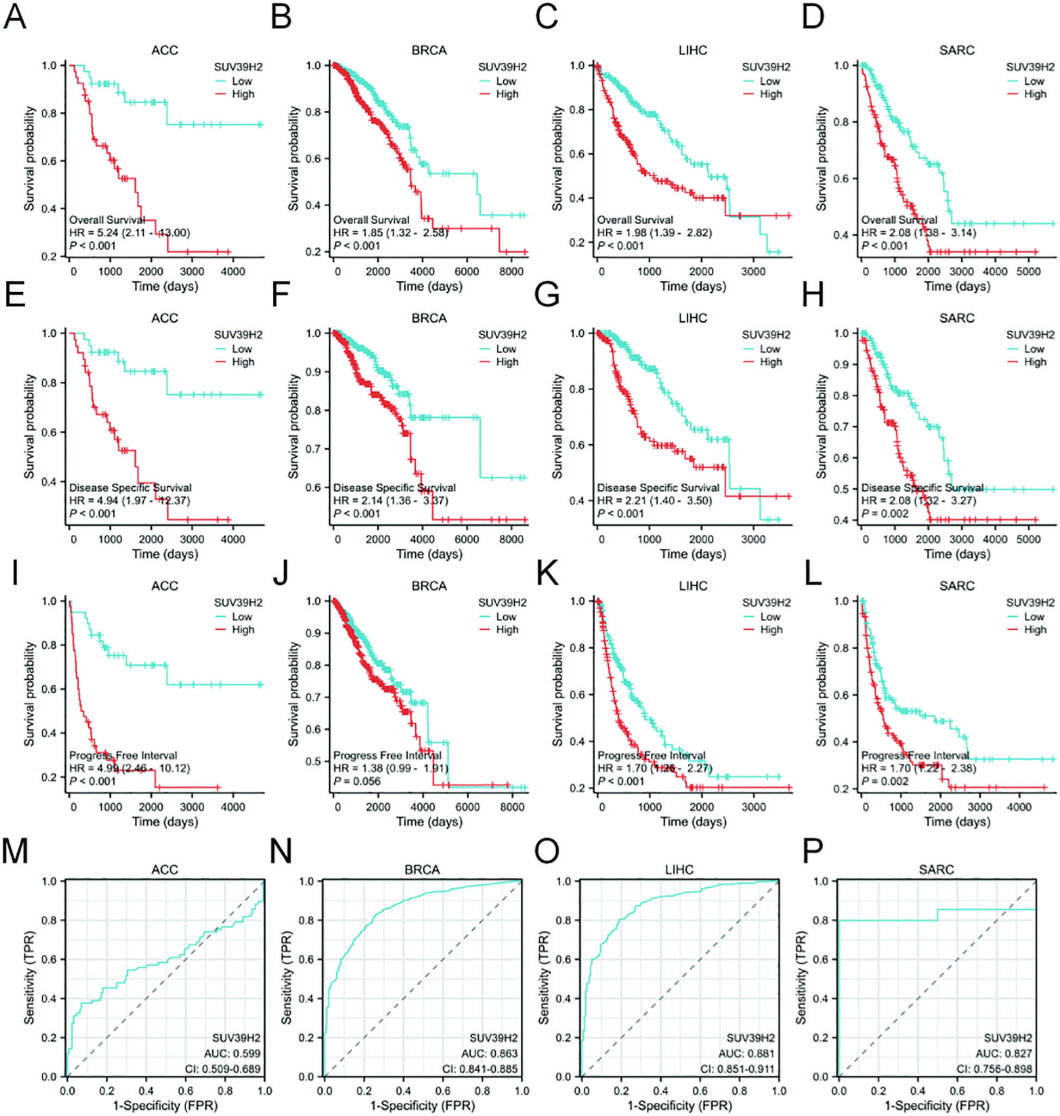
Prognostic and diagnostic value of SUV39H2 in ACC, BRCA, LIHC, and SARC. **(A–L)** Kaplan–Meier survival analyses of OS, DSS, and PFI based on SUV39H2 expression levels. **(M–P)** ROC curves assessing the diagnostic performance of SUV39H2 in four cancer types.

To examine SUV39H2 expression in tumor tissues, the Xiantao online tool was used to analyze mRNA levels in multiple tumor types from the TCGA-GTEX database (**Figure 1A**), along with paired adjacent normal tissues from the TCGA database (**Figure 1B**). The results indicated that SUV39H2 was highly expressed in a range of tumors and similarly elevated in paired samples, suggesting its involvement in tumor progression. Subsequent prognostic analysis (**Figures 2A-L**) showed that SUV39H2 expression was associated with prognosis in ACC, BRCA, LIHC, and SARC, with high SUV39H2 expression linked to poorer OS, DSS, and PFI. Although the difference in PFI was not significant for BRCA, the overall trend indicated that patients with lower SUV39H2 expression had better outcomes. These findings suggest that SUV39H2 may play a significant role in the progression of these four cancers. ROC curve analysis of patient samples (**Figures 2M-P**) showed that SUV39H2 could serve as a promising clinical diagnostic marker in BRCA, LIHC, and SARC.

### SUV39H2 expression in BRCA and LIHC

**Figure 3:**
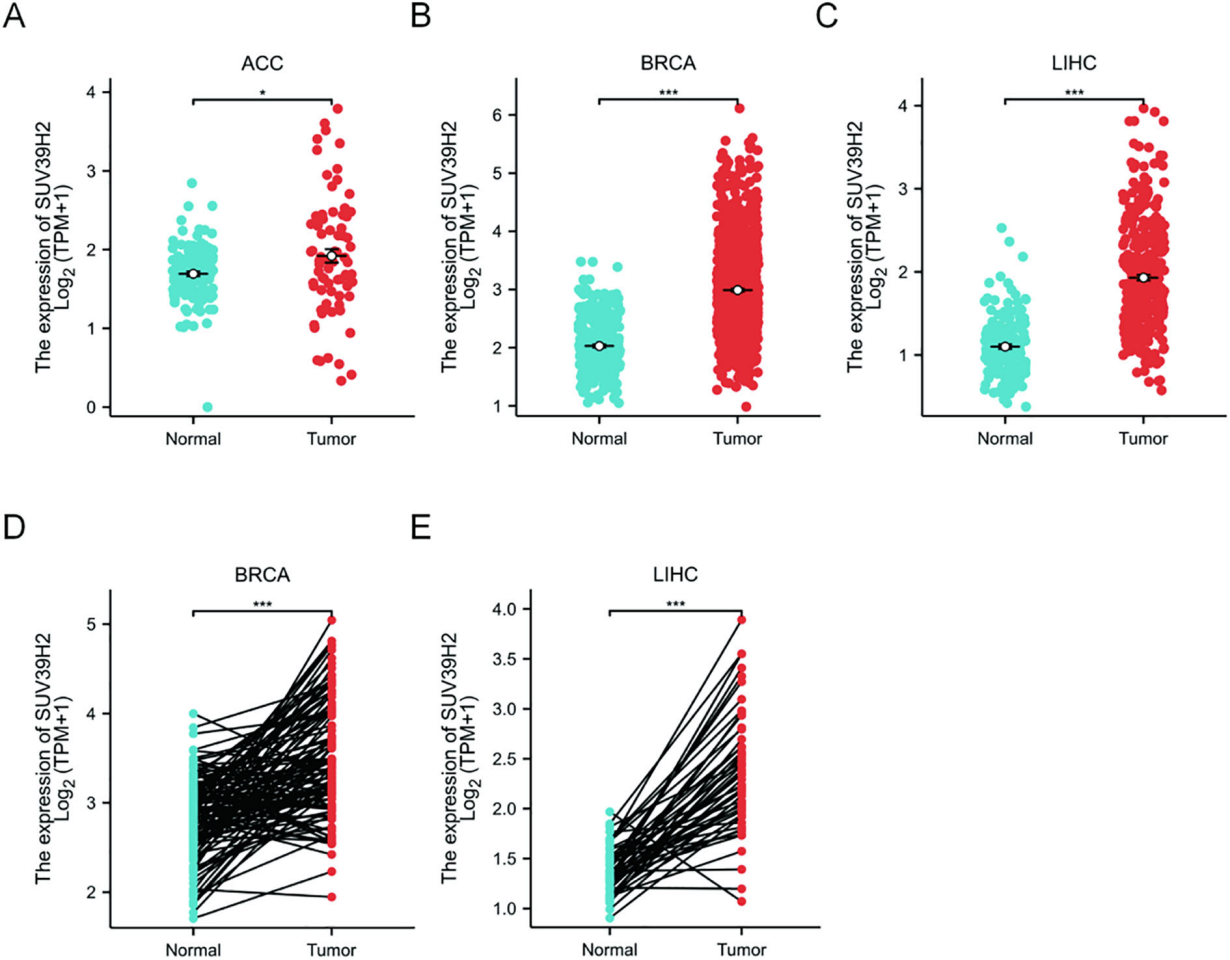
SUV39H2 is upregulated in BRCA and LIHC tumor tissues. **(A–C)** Comparison of SUV39H2 mRNA expression in tumor and normal tissues for ACC **(A)**, BRCA **(B)**, and LIHC **(C)** using TCGA-GTEx unpaired data. All three cancer types exhibited significantly higher SUV39H2 expression in tumor tissues. **(D)** Paired analysis of SUV39H2 expression in BRCA samples showed significantly elevated levels in tumors compared to adjacent normal tissues. **(E)** Paired comparison in LIHC confirmed similar upregulation of SUV39H2 in tumor samples. Statistical significance was determined using the Wilcoxon test; P < 0.05 (*), P < 0.001 (***).

Analysis of ACC, BRCA, LIHC, and SARC samples from TCGA-GTEX confirmed significant upregulation of SUV39H2 in all four tumor types (**Figures 3A–C**). Due to the lack of paired samples for ACC and SARC in the TCGA dataset, paired sample analysis focused on BRCA and LIHC (**Figures 3D, E**), revealing significantly elevated SUV39H2 expression in these two tumor types. These findings suggest that SUV39H2 may be essential in BRCA and LIHC.

### Association of SUV39H2 expression with clinical stages in BRCA and LIHC

**Figure 4:**
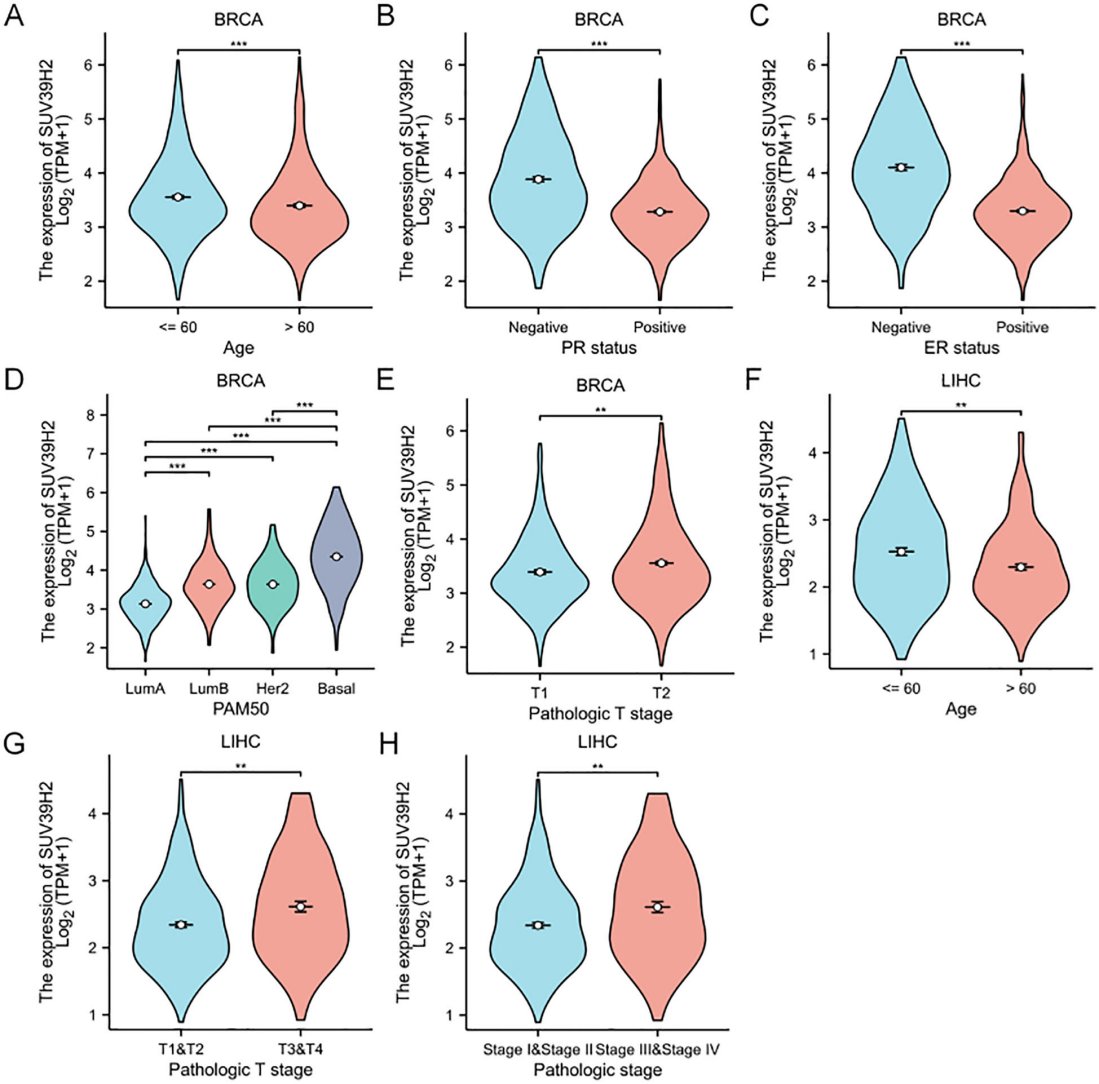
SUV39H2 expression is associated with clinical and molecular characteristics in BRCA and LIHC. **(A)** In BRCA, SUV39H2 expression was significantly higher in patients aged ≤60 years than in older patients. **(B, C)** SUV39H2 levels were significantly elevated in PR-negative (b) and ER-negative (c) BRCA tumors compared to hormone receptor–positive tumors. **(D)** Among PAM50-defined molecular subtypes, SUV39H2 expression was highest in the basal-like group, with lower expression in Luminal A, Luminal B, and HER2-enriched subtypes. **(E)** SUV39H2 expression was higher in T2-stage BRCA tumors than in T1-stage tumors, suggesting a link to tumor growth. **(F)** In LIHC, patients >60 years showed increased SUV39H2 expression compared to those ≤60 years. **(G)** SUV39H2 expression was elevated in advanced LIHC T stages (T3&T4) compared to early stages (T1&T2). **(H)** Higher SUV39H2 levels were observed in LIHC patients with pathological stage III–IV versus stage I–II. P < 0.01 (**), P < 0.001 (***).

Clinical stage analysis in TCGA BRCA and LIHC samples showed that SUV39H2 expression was higher in patients aged ≤60 years (**Figures 4A, F**), suggesting a possible link to more aggressive disease in younger patients. In BRCA, SUV39H2 was more highly expressed in PR- and ER-negative samples and was associated with molecular subtypes (**Figures 4B-D**). SUV39H2 expression increased in T2 compared to T1 in BRCA (**Figure 4E**), suggesting a role in tumor growth. In LIHC, SUV39H2 expression also increased in later T stages (**Figures 4G, H**). These results indicate that SUV39H2 expression is associated with age, T stage, and molecular subtype in BRCA and LIHC.

### KEGG and GO analyses of SUV39H2 in BRCA and LIHC

**Figure 5:**
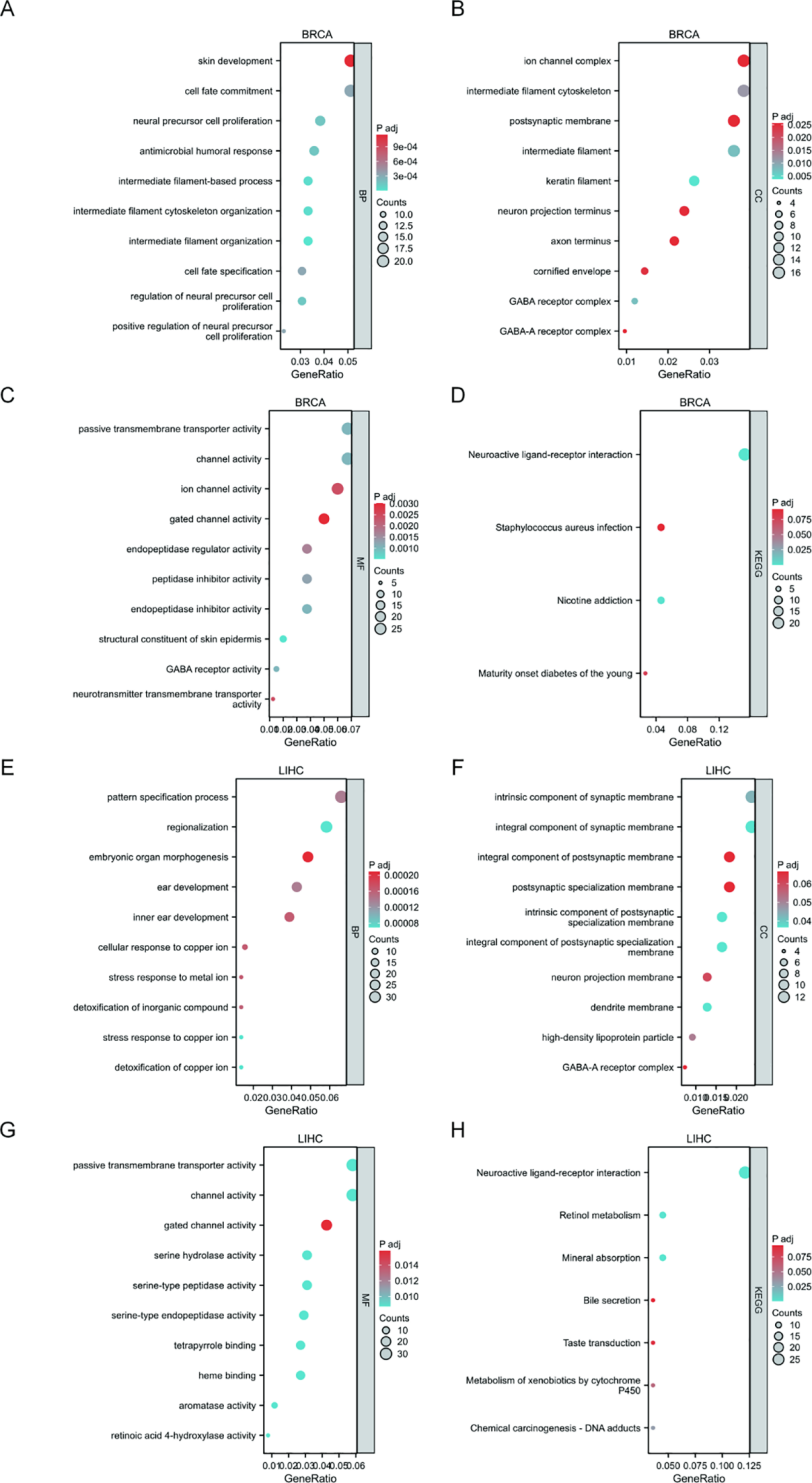
Functional enrichment analysis of SUV39H2-related genes in BRCA and LIHC. **(A, E)** GO enrichment in the biological process (BP) category showed that SUV39H2-associated genes in BRCA **(A)** and LIHC **(E)** were involved in pathways including cell development, epithelial cell proliferation, and differentiation. **(B, F)** In the cellular component (CC) category, SUV39H2 was associated with synaptic membrane, ion channel complexes, and neuron projections in BRCA **(B)** and LIHC **(F)**. **(C, G)** The molecular function (MF) analysis revealed enrichment in ion channel activity, transcription factor binding, and peptidase regulator activity in both BRCA **(C)** and LIHC **(G)**. **(D, H)** KEGG pathway enrichment indicated that SUV39H2 may act through neuroactive ligand–receptor interaction, GABAergic synapse, and retinol metabolism signaling in BRCA **(D)** and LIHC **(H)**.

To further assess the role of SUV39H2, KEGG and GO analyses were performed using Xiantao tools on TCGA-BRCA and TCGA-LIHC samples (**Figure 5**). In the BP category (**Figures 5A, E**), SUV39H2 expression was linked to cell development, cell fate commitment, and structural organization in BRCA, as well as to cell development in LIHC, suggesting a role in differentiation. In the CC category, SUV39H2 was associated with cellular communication in both cancers (**Figures 5B, F**). MF analysis suggested that SUV39H2 might regulate peptidase activity via cell membrane receptors in BRCA and LIHC (**Figures 5C, G**). KEGG analysis indicated that SUV39H2 may promote tumor progression through the neuroactive ligand-receptor interaction pathway (**Figures 5D, H**). Together, these results suggest that SUV39H2 may regulate peptidase activity and promote tumor cell development and differentiation through this pathway.

### Association of SUV39H2 with immune infiltration in BRCA and LIHC

**Figure 6:**
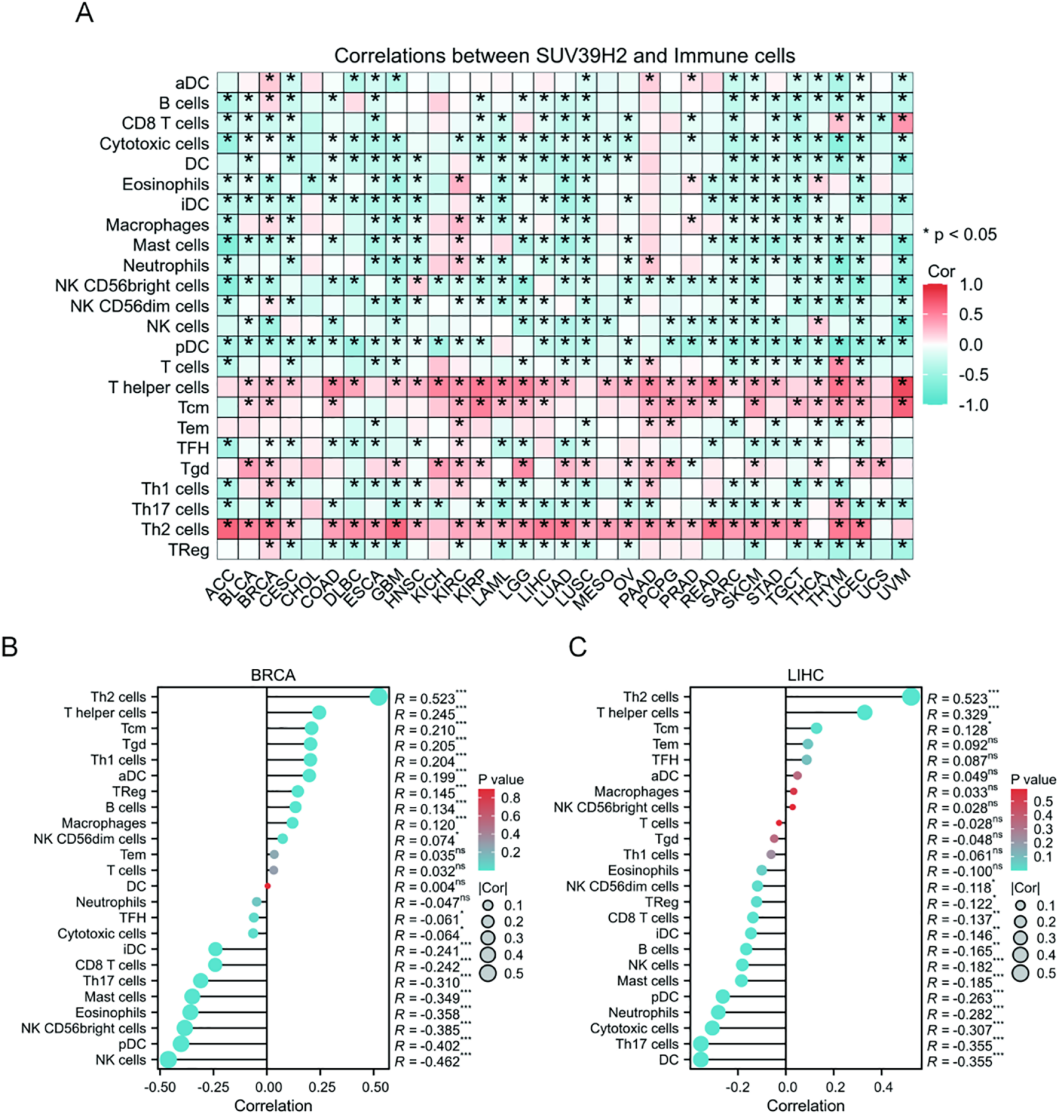
Correlation of SUV39H2 expression with immune cell infiltration in BRCA and LIHC. **(A)** Heatmap showing correlations between SUV39H2 expression and infiltration levels of 24 immune cell types across various tumor types. Red and blue indicate positive and negative correlations, respectively. **(B)** In BRCA, SUV39H2 expression positively correlated with Th2 and T helper cells, and negatively correlated with CD8^+^ T cells, NK cells, and pDCs. **(C)** Similar patterns were observed in LIHC, with SUV39H2 expression showing positive correlation with immunosuppressive cell subsets and inverse correlation with cytotoxic effectors. P > 0.05 (ns), P < 0.05 (*), P < 0.01 (**), P < 0.001 (***).

Analysis of TCGA samples showed that SUV39H2 expression correlated with various immune cell types (**Figure 6A**), with subtle differences across tumors. In BRCA and LIHC (**Figures 6B, C**), SUV39H2 expression was positively correlated with Th2 and T helper cells, suggesting a role in promoting tumor growth and inflammation. In contrast, SUV39H2 expression was negatively correlated with CD8 T cells, NK cells, and pDCs, indicating possible suppression of immune responses. These results suggest that SUV39H2 may promote tumor progression by inducing inflammation and suppressing immune cell infiltration.

### Single-cell RNA sequencing analysis of SUV39H2

**Figure 7:**
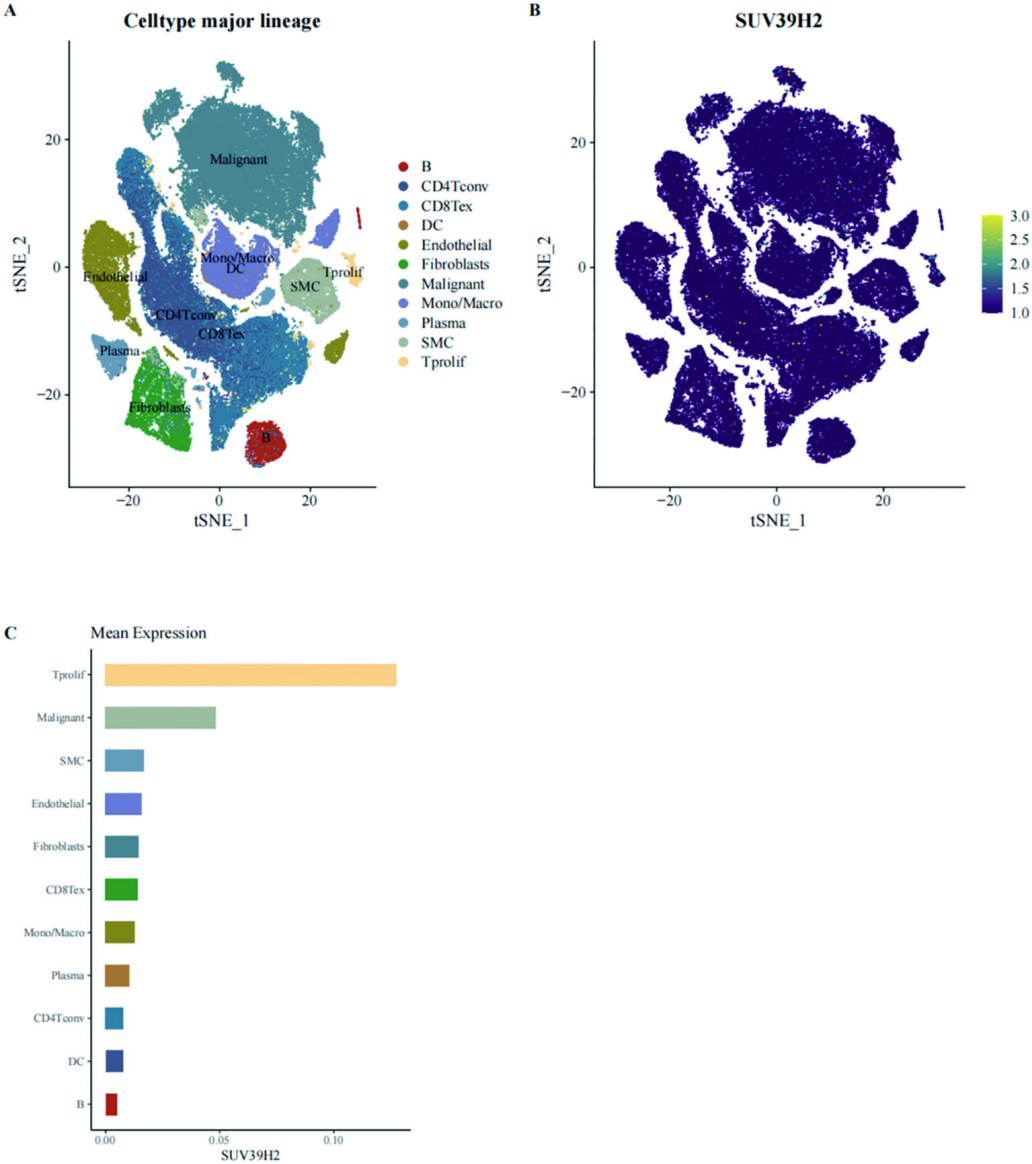
Single-cell transcriptomic analysis of SUV39H2 expression in BRCA. **(A)** t-SNE plot of major cell populations in BRCA samples from the GSE176078 dataset, including malignant, immune, and stromal lineages. **(B)** SUV39H2 expression mapped onto the t-SNE projection revealed predominant localization in malignant cell clusters. **(C)** Mean expression of SUV39H2 across cell types confirmed its highest expression in malignant and proliferating tumor cells.

Previous TCGA transcriptome analyses revealed SUV39H2 overexpression in BRCA. To validate this at the single-cell level, BRCA samples from GSE176078 were analyzed. Dimensionality reduction and clustering were performed (**Figure 7A**), and SUV39H2 expression was examined (**Figures 7B, C**). SUV39H2 expression was significantly higher in malignant cells compared to endothelial cells, confirming its overexpression in BRCA tumor cells.

### SUV39H2 expression and clinical staging in TNBC

**Figure 8:**
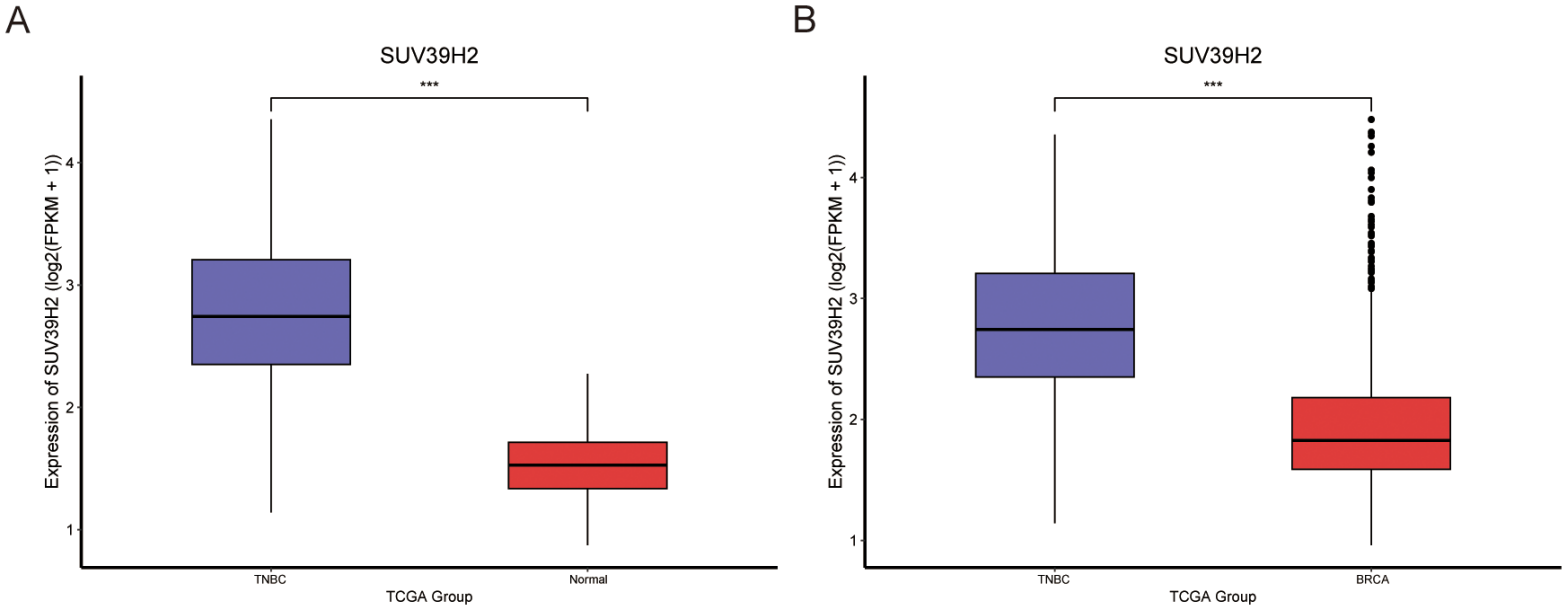
SUV39H2 expression is elevated in TNBC compared to normal tissues and non-TNBC BRCA subtypes. SUV39H2 expression in TNBC samples was significantly higher than in normal breast tissues. SUV39H2 levels were also elevated in TNBC compared to non-TNBC BRCA subtypes, suggesting subtype-specific upregulation. P < 0.001 (***).

**Figure 9:**
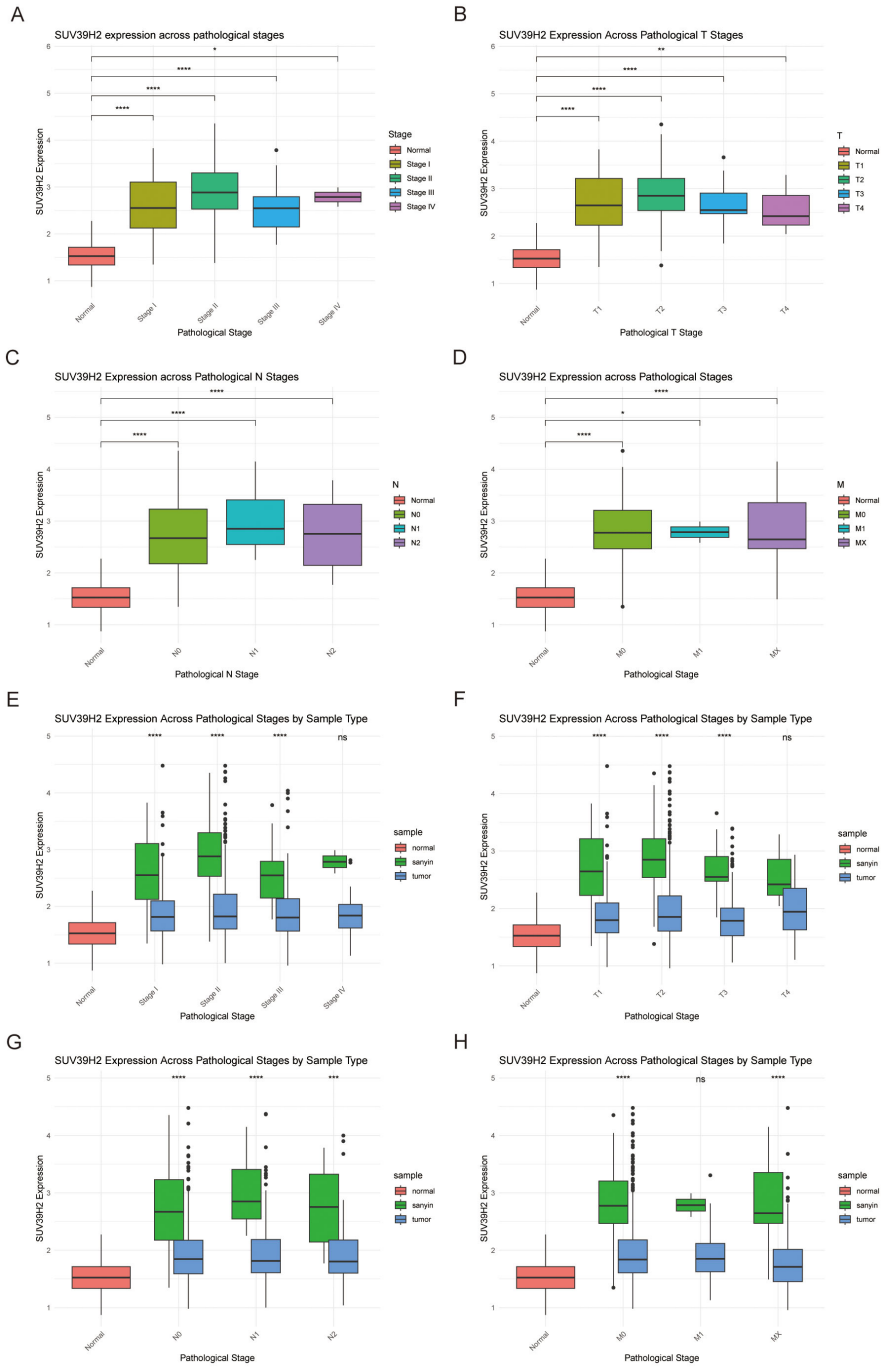
SUV39H2 expression across pathological and TNM stages in TNBC and its comparison with BRCA subtypes. **(A)** SUV39H2 expression across pathological stages (Stage I–IV) in TNBC samples, showing significantly higher expression in Stage II and III compared to Stage I. **(B)** SUV39H2 expression across T stages (T1–T4) within TNBC, indicating a positive association with tumor size progression. **(C)** Expression of SUV39H2 across N stages (N0–N3) in TNBC, with elevated levels in lymph node–positive tumors. **(D)** Comparison of SUV39H2 expression across M stages (M0 vs M1) in TNBC, suggesting stable overexpression regardless of metastatic status. **(E)** SUV39H2 expression across pathological stages (I–IV) compared between TNBC, BRCA, and normal samples, showing consistently higher levels in TNBC across all stages. **(F)** T stage–based comparison of SUV39H2 expression between TNBC, BRCA, and normal samples, revealing significant upregulation in TNBC at both early and advanced stages. **(G)** N stage–based SUV39H2 expression comparison among TNBC, BRCA, and normal tissues, further supporting subtype-specific elevation. **(H)** SUV39H2 expression across M stages stratified by TNBC, BRCA, and normal groups, confirming sustained upregulation in TNBC irrespective of metastasis status.P > 0.05 (ns), P < 0.05 (*), P < 0.01 (**), P < 0.001 (***), P < 0.0001 (****).

Analysis of BRCA subtypes revealed further elevated SUV39H2 expression in the Basal subtype. Using R, 115 TNBC samples from TCGA were identified. SUV39H2 expression was significantly higher in TNBC samples compared to normal tissues (**Figure 8A**) and other BRCA subtypes (**Figure 8B**). SUV39H2 expression was also elevated in all clinical and TNM stages of TNBC compared to normal tissues (**Figures 9A-D**). When comparing TNBC to other BRCA subtypes, SUV39H2 expression remained higher (**Figures 9E-H**). Although some comparisons were not significant, likely due to limited sample sizes, the overall trend persisted. These findings suggest that SUV39H2 is consistently upregulated in TNBC and may contribute to its high metastatic and recurrent potential.

### SUV39H2 knockdown inhibits TNBC cell growth and metastasis

**Figure 10:**
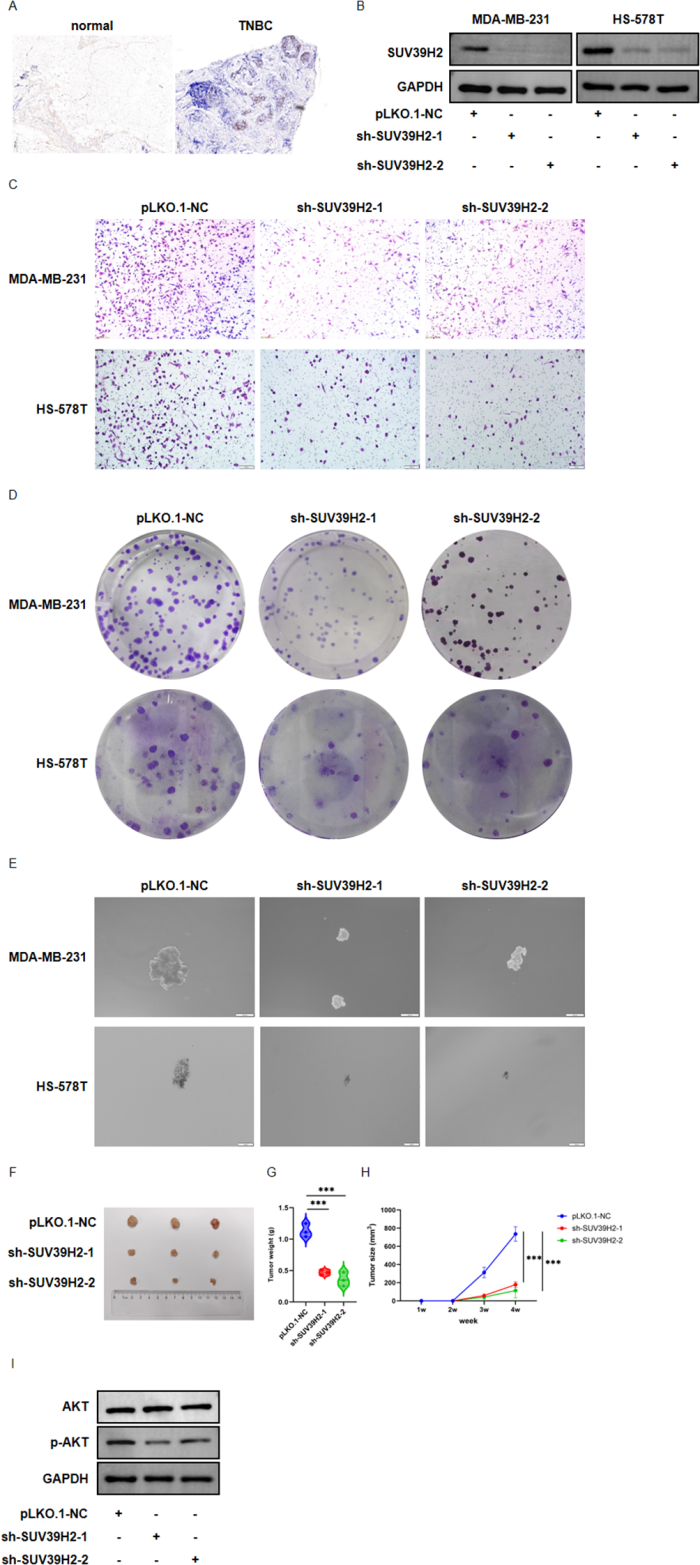
SUV39H2 knockdown inhibits proliferation, stemness, invasion, and tumorigenicity of TNBC cells. **(A)** Immunohistochemistry (IHC) staining of SUV39H2 in clinical TNBC tumor tissues and adjacent normal tissues, showing stronger nuclear expression in tumor samples. **(B)** Western blot analysis confirming SUV39H2 knockdown efficiency in MDA-MB-231 and Hs-578T cells transduced with two independent shRNAs (sh-SUV39H2–1 and sh-SUV39H2-2) compared to control (pLKO.1-NC). **(C)** Transwell invasion assay showing decreased invasive capacity in SUV39H2 knockdown cells relative to control. **(D)** Colony formation assay indicating reduced clonogenic potential upon SUV39H2 silencing. **(E)** Sphere formation assay demonstrating impaired tumor sphere formation and stemness after SUV39H2 knockdown. **(F)***In vivo* tumor xenograft model using nude mice, showing decreased tumor size and volume in both SUV39H2 knockdown groups compared to control. **(G, H)** Tumor weight and size measurements of the subcutaneous xenografts. **(I)** Western blot analysis detection of AKT phosphorylation level changes following SUV39H2 knockdown.

To investigate the functional role of SUV39H2, immunohistochemistry was performed on five pairs of TNBC patient samples, confirming SUV39H2 overexpression (**Figure 10A**). To investigate the role of SUV39H2 in TNBC cell lines, we selected MDA-MB-231 and Hs-578T cells for subsequent experiments. After establishing the sh-SUV39H2 knockdown cell lines, we assessed the changes in its expression levels by performing Western blotting (**Figure 10B**). Transwell, colony formation, sphere formation, and *in vivo* tumorigenesis assays (**Figures 10C-F**) showed that SUV39H2 knockdown significantly inhibited cell proliferation, stemness, metastasis, and tumor formation in MDA-MB-231 cells. Additionally, we weighed and measured the size and volume of the subcutaneous xenografts, and the results further validated the inhibitory effect of SUV39H2 knockdown on the growth of MDA-MB-231 cells (**Figures 10G, H**). Finally, in order to explore the mechanism of SUV39H2, we detected changes in AKT phosphorylation levels and found that reduced SUV39H2 expression significantly inhibited AKT phosphorylation (**Figure 10I**).

## Discussion

SUV39H2 was found to be significantly overexpressed in multiple tumor types, including ACC, BRCA, LIHC, and SARC, with this elevated expression also evident in matched adjacent normal tissues. These observations align with previous studies that identified SUV39H2 as a histone methyltransferase responsible for H3K9me3 modification, thereby promoting chromatin silencing and transcriptional repression (38). Such overexpression likely contributes to the silencing of tumor suppressor genes and thus facilitates tumorigenesis and progression. Moreover, elevated SUV39H2 expression was strongly correlated with poor prognosis, including worse OS, DSS, and PFI, underscoring its potential role in tumor development (39). InIn BRCA and LIHC, SUV39H2 overexpression was further associated with clinical features such as younger patient age, advanced T stage, and molecular subtypes, suggesting that SUV39H2 may contribute to more aggressive tumor phenotypes (40).

SUV39H2 expression was also associated with clinical features such as younger patient age, advanced T stage, and molecular subtypes in BRCA and LIHC. In BRCA, higher SUV39H2 expression was observed in PR- and ER-negative patients and in the Basal-like subtype, implicating SUV39H2 in more aggressive tumor phenotypes (40). In TNBC samples, SUV39H2 expression was consistently elevated across all clinical stages, implicating a potential role in metastasis and recurrence (40). These findings suggest that SUV39H2 may promote malignancy in younger patients and more aggressive subtypes by modulating pathways involved in proliferation and migration.

Functional annotation through GO and KEGG analyses indicated that SUV39H2 may regulate processes including cell development, differentiation, and communication in BRCA and LIHC, and may involve membrane receptor activity and the neuroactive ligand-receptor interaction signaling pathway (41, 42). These data imply a multifaceted role for SUV39H2 in supporting tumor cell proliferation, differentiation, and invasion. Furthermore, immune infiltration analyses revealed a positive correlation between SUV39H2 expression and pro-inflammatory immune cells such as Th2 cells, along with a negative correlation with anti-tumor immune populations, including CD8+ T cells, NK cells, and pDCs. This pattern suggests that SUV39H2 may promote an immunosuppressive microenvironment, enabling immune evasion and thereby accelerating tumor progression (43).

Single-cell RNA sequencing data further confirmed the elevated expression of SUV39H2 in malignant BRCA cells, reinforcing its role as a tumor-associated factor (44). Functional assays demonstrated that knockdown of SUV39H2 in TNBC cell lines significantly suppressed proliferation, migration, stemness, and *in vivo* tumorigenicity. These results validate the oncogenic function of SUV39H2 and highlight its potential as a therapeutic target.

Despite these comprehensive analyses based on TCGA, GTEX, and GEO datasets and experimental validations, certain limitations remain. Immune infiltration and pathway analyses were based on bioinformatic predictions and warrant further mechanistic validation (45). Sample sizes for some subgroups, such as different TNBC clinical stages, were limited and may have reduced statistical power; expanding the cohort through multi-center cooperation will be essential to improve data reliability. Functional validation was confined to TNBC models, and whether SUV39H2 plays similar roles in other tumor subtypes remains to be verified. Importantly, while our findings support SUV39H2 as a potential biomarker in TNBC, its clinical applications—such as early detection, risk stratification, prognostic assessment, or guiding targeted therapy—require validation in large, independent patient cohorts before translation into routine practice. Therefore, SUV39H2 should currently be regarded as a candidate marker with translational potential, rather than a definitive diagnostic biomarker.

## Data Availability

The raw data supporting the conclusions of this article will be made available by the authors, without undue reservation.
