# Supplementary material for: Role of SUV39H2 in shaping the malignant phenotype of triple-negative breast cancer
Source: Front Oncol. 2026 Jan 2;15:1679640. doi: 10.3389/fonc.2025.1679640 (PMC12807893; doi:10.3389/fonc.2025.1679640)
Supplement: Supplementary file 1 [file DataSheet1.pdf]

Table S1

| Name | Abbreviation |
|---|---|
| Bladder Urothelial Carcinoma | BLCA |
| Breast invasive carcinoma | BRCA |
| Cholangiocarcinoma | CHOL |
| Colon adenocarcinoma | COAD |
| Esophageal carcinoma | ESCA |
| Head and Neck squamous cell carcinoma | HNSC |
| Kidney renal clear cell carcinoma | KIRC |
| Kidney renal papillary cell carcinoma | KIRP |
| Liver hepatocellular carcinoma | LIHC |
| Lung adenocarcinoma | LUAD |
| Lung squamous cell carcinoma | LUSC |
| Prostate adenocarcinoma | PRAD |
| Rectum adenocarcinoma | READ |
| Stomach adenocarcinoma | STAD |
| Thyroid carcinoma | THCA |
| Uterine Corpus Endometrial Carcinoma | UCEC |
